# Supplementary material for: Allosteric control of an asymmetric transduction in a G protein-coupled receptor heterodimer
Source: eLife. 2017 Aug 10;6:e26985. doi: 10.7554/eLife.26985 (PMC5582870; doi:10.7554/eLife.26985)
Supplement: Figure 1—source data 1. — Intracellular Ca2+ responses mediated by the indicated subunits upon stimulation with increasing concentrations of glutamate. 2X and 4X indicated subunits carrying the F756S (mGlu2) or F781S (mGlu4) mutation preventing G protein activation. Data represent the means ± SEM of (n) independent experiments. N.D., not determined. [file elife-26985-fig1-data1.docx]

|  | | | |  |  |
| --- | --- | --- | --- | --- | --- |
|  | pEC50 |  | Max  (% of control) |  | Max  (% of mGlu2-2) |
| 2-2 | 4.89 ± 0.07 (5) |  | 100 |  | 100 |
| 2^X^-2 | 4.50 ± 0.16 (4) |  | 49.8 ± 2.8 (4) |  |  |
| 2^X^-2^X^ | N.D. |  | 2.3 ± 1.0 (4) |  |  |
| 4-4 | 4.79 ± 0.15 (7) |  | 100 |  | 43.1 ± 6.7 (3) |
| 4^X^-4 | 4.56 ± 0.08 (6) |  | 70.6 ± 4.7 (6) |  |  |
| 4^X^-4^X^ | N.D. |  | 3.2 ± 0.9 (4) |  |  |
| 2-4 | 5.37 ± 0.08 (11) |  | 100 |  | 98.6 ± 7.8 (6) |
| 2^X^-4 | 5.48 ± 0.16 (5) |  | 88.8 ± 9.2 (5) |  |  |
| 2-4^X^ | N.D. |  | 2.8 ± 3.3 (6) |  |  |

**Figure 1-source data file 1**: **Glutamate potency and relative efficacy at the indicated heterodimers**

Intracellular Ca^2+^ responses mediated by the indicated receptor dimers upon stimulation with increasing concentrations of glutamate. 2^X^ and 4^X^ indicated subunits carrying the F756S (mGlu2) or F781S (mGlu4) mutation preventing G protein activation. Maximal responses were calculated as the difference between the maximal response and the basal, and normalized to that determined with the control. Data represent the means ± SEM of (n) independent experiments. N.D., not determined.
