# Supplementary material for: Allosteric control of an asymmetric transduction in a G protein-coupled receptor heterodimer
Source: eLife. 2017 Aug 10;6:e26985. doi: 10.7554/eLife.26985 (PMC5582870; doi:10.7554/eLife.26985)
Supplement: Figure 4—source data 1. — Intracellular Ca2+ response mediated by indicated subunits upon stimulation with glutamate (1 mM) and inhibited by increasing concentration of MNI137. Data represent the means ± SEM of (n) independent experiments. N.D., not determined. [file elife-26985-fig4-data1.docx]

|  | | | | |
| --- | --- | --- | --- | --- |
|  |  | pIC50 |  |  |
| Glutamate  (1mM) | 2-2 | 6.79 ± 0.13 (3) |  |  |
|  | 4-4 | N.D. |  |  |
|  | 2-4 | 6.56 ± 0.05 (6) |  |  |
|  | 2^X^-4 | 6.58 ± 0.10 (3) |  |  |

**Figure 4-source data file 1: MNI137 potency at the indicated mGlu dimers.**

Intracellular Ca^2+^ response mediated by indicated subunits upon stimulation with glutamate (1mM) and inhibited by increasing concentration of MNI137. Data represent the means ± SEM of (n) independent experiments. N.D., not determined.
