# Supplementary material for: Allosteric control of an asymmetric transduction in a G protein-coupled receptor heterodimer
Source: eLife. 2017 Aug 10;6:e26985. doi: 10.7554/eLife.26985 (PMC5582870; doi:10.7554/eLife.26985)
Supplement: Figure 4—source data 2. — Inositol phosphate (IP) accumulation in cells expressed indicated subunits, which have constitutive activation, upon inhibition with increasing concentration of MN137. Data represent the means ± SEM of (n) independent experiments. N.D., not determined. [file elife-26985-fig4-data2.docx]

|  | | | | |  |  |  |
| --- | --- | --- | --- | --- | --- | --- | --- |
|  | pIC50 |  |  |  | |  |  |
| 2^C^-2^C^ | 6.30 ± 0.14 (3) |  |  |  | |  |  |
| 4^C^-4^C^ | N.D. |  |  |  | |  |  |
| 2^C^-4^C^ | 6.19 ± 0.11 (4) |  |  |  | |  |  |
| 2^C-X^-4^C^ | 6.29 ± 0.20 (3) |  |  |  | |  |  |

**Figure 4-source data file 2: MNI137 potency at the indicated mGlu dimers.**

Inositol phosphate (IP) accumulation in cells expressed indicated subunits, which have constitutive activation, upon inhibition with increasing concentration of MN137. Data represent the means ± SEM of (n) independent experiments. N.D., not determined.
