# Supplementary material for: Allosteric control of an asymmetric transduction in a G protein-coupled receptor heterodimer
Source: eLife. 2017 Aug 10;6:e26985. doi: 10.7554/eLife.26985 (PMC5582870; doi:10.7554/eLife.26985)
Supplement: Figure 4—source data 3. — Intracellular Ca2+ response mediated by indicated heterodimer upon stimulation with DCG-IV (30 μM), L-AP4 (30 μM) or DCG-IV (30 μM)+L-AP4 (30 μM) and inhibited by increasing concentration of MNI137. Data represent the means ± SEM of (n) independent experiments. N.D., not determined. [file elife-26985-fig4-data3.docx]

|  | | | | |
| --- | --- | --- | --- | --- |
|  |  | pIC50 |  |  |
| DCG-IV (30µM) | 2-4 | 7.05 ± 0.05 (4) |  |  |
| L-AP4 (30µM) | 2-4 | 7.19 ± 0.06 (4) |  |  |
| DCG-IV (30µM)  +L-AP4 (30µM) | 2-4 | 6.00 ± 0.49 (3) |  |  |

**Figure 4-source data file 3**: MNI137 potency at the indicated heterodimers

Intracellular Ca^2+^ response mediated by indicated heterodimer upon stimulation with DCG-IV (30μM), L-AP4 (30μM) or DCG-IV (30μM) + L-AP4 (30μM) and inhibited by increasing concentration of MNI137. Data represent the means ± SEM of (n) independent experiments. N.D., not determined.
