# Supplementary material for: Allosteric control of an asymmetric transduction in a G protein-coupled receptor heterodimer
Source: eLife. 2017 Aug 10;6:e26985. doi: 10.7554/eLife.26985 (PMC5582870; doi:10.7554/eLife.26985)
Supplement: Figure 5—source data 1. — Intracellular Ca2+ response mediated by indicated subunits upon stimulation with increasing concentration of glutamate with/without optoGluNAM4.1 (30μM) or LY487379 (10 μM). Data represent the means ± SEM of (n) independent experiments. N.D., not determined. [file elife-26985-fig5-data1.docx]

|  | | |
| --- | --- | --- |
|  |  | pEC50 |
| 2-4 |  | 5.37 ± 0.08 (11) |
|  | OptoGluNAM4.1 (30µM) | 5.83 ± 0.12 (2) |
|  | LY487379 (10µM) | 5.62 ± 0.16 (3) |
| 2-4^X^ |  | N.D. |
|  | OptoGluNAM4.1 (30µM) | 4.53 ± 0.77 (2) |
|  | LY487379 (10µM) | 5.81 ± 0.20 (3) |
| 2^X^-4 |  | 5.48 ± 0.16 (5) |
|  | LY487379 (10µM) | 5.58 ± 0.15 (3) |

**Figure 5-source data file 1: Glutamate potency at the indicated heterodimers and in the presence or absence of the indicated allosteric modulators.**

Intracellular Ca^2+^ response mediated by indicated subunits upon stimulation with increasing concentration of glutamate with/without optoGluNAM4.1 (30μM) or LY487379 (10μM). Data represent the means ± SEM of (n) independent experiments. N.D., not determined.
